# Large cell neuroendocrine carcinoma of the ovary: a case report and a brief review of the literature

**DOI:** 10.1186/1477-7819-12-314

**Published:** 2014-10-15

**Authors:** Eun Young Ki, Jong Sup Park, Keun Ho Lee, Seog Nyeon Bae, Soo Young Hur

**Affiliations:** Department of Obstetrics and Gynecology, Seoul St. Mary’s Hospital, The Catholic University of Korea, Banpodaero, Seocho-Gu, Seoul, Korea

**Keywords:** Ovary, Neuroendocrine carcinoma, Non-small cell, Large cell

## Abstract

Large cell neuroendocrine carcinoma (LCNC) of the ovary, or ovarian undifferentiated non-small cell carcinoma of neuroendocrine type, is a rare entity that is frequently associated with ovarian surface epithelial tumors. Few cases have been reported in the literature. LCNC is an aggressive tumor with tendency to present at advanced stages and to cause death after a short postoperative duration. We report three cases of LCNC diagnosed histopathologically. Immunohistochemically, the tumor cells were positive for chromogranin A, NSE, CD56, and pancytokeratin. The patients were treated postoperatively with combination chemotherapy. Due to the rarity of LCNC, the general consensus on standard therapy is not established. Although most patients are at stage I, the biological aggressiveness and poor prognosis of the tumors have been reported in previous reports despite extensive surgery and chemotherapy.

## Background

Ovarian neoplasms associated with hormone production and secretion include sex-cord-stromal and germ cell tumors [1]. Another tumor showing endocrine features is small cell carcinoma, which is divided into hypercalcemic and pulmonary types [2]. According to the World Health Organization (WHO), undifferentiated non-small cell carcinoma of neuroendocrine type is synonymous with large cell neuroendocrine carcinoma (LCNC) [3, 4]. LCNC is a rare cancer, and 27 cases have been reported in the literature so far [2–7]. The clinical behaviors of LCNC are aggressive and show poor prognosis despite being diagnosed in the early stages [2, 8, 9]. Most of the reported LCNCs are associated with teratoma or epithelial tumor, such as serous and mucinous tumors [10–12].

We report three cases of large cell neuroendocrine carcinoma of the ovary with a literature review.

## Case presentation

### Case 1

**Figure 1:**
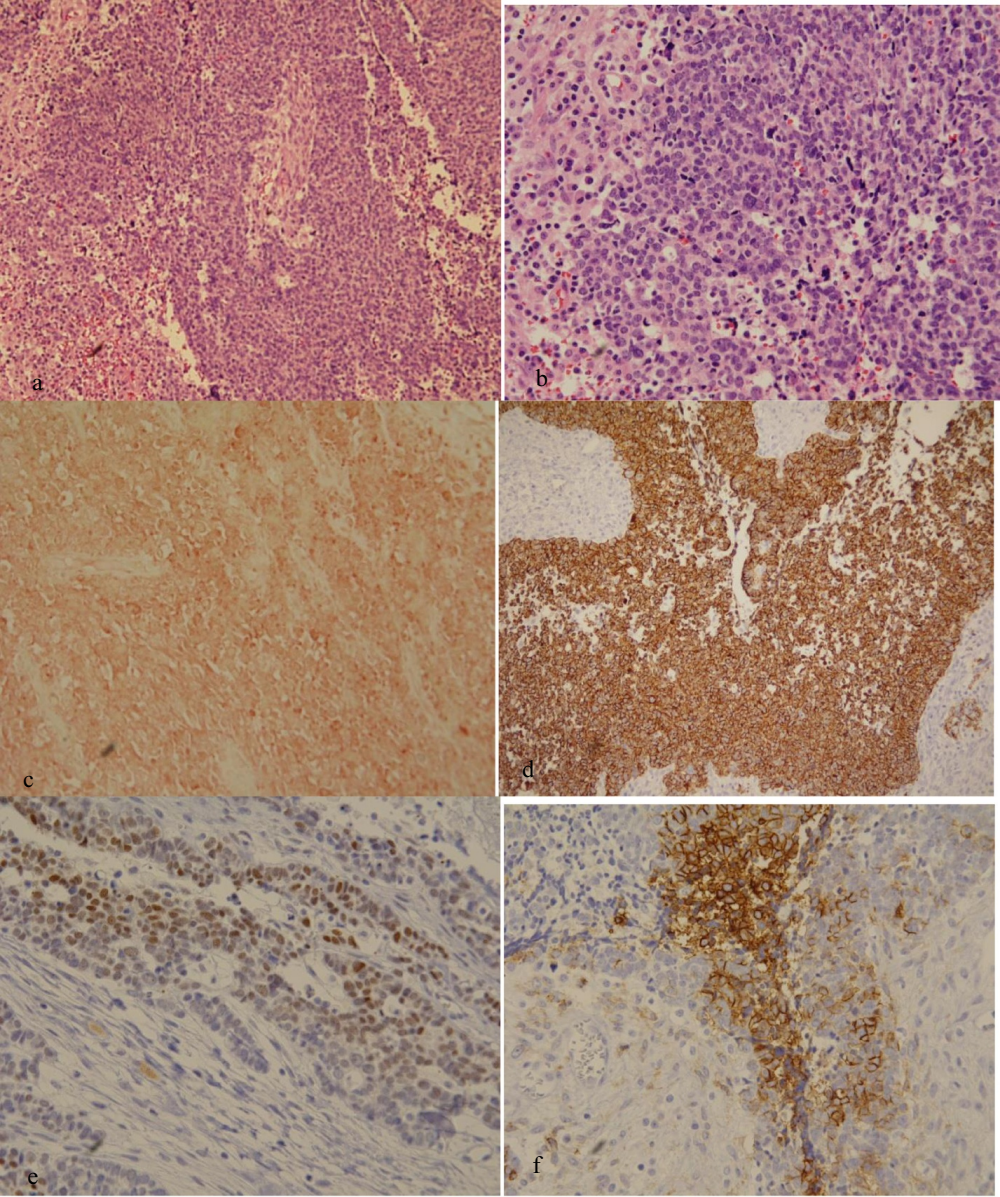
**Microscopic pictures. (a)** Neuroendocrine carcinoma: low-power field shows solid sheets (H&E, ×40). **(b)** Cells with larger vesicular nuclei: prominent nucleoli, and mitotic activity (H&E, ×400). **(c)** Chromogranin A is expressed in a neuroendocrine carcinoma (×400). **(d)** Pancytokeratin is expressed in a neuroendocrine carcinoma (×400). **(e)** NSE is expressed focally in a neuroendocrine carcinoma (×400). **(f)** CD56 is expressed in a neuroendocrine carcinoma (×400).

A 77-year old woman visited our clinic with a 1-month history of abdominal distension and discomfort. She had been diagnosed with coronary artery disease prior to this presentation. Physical examination showed ascites and firm/fixed mass in the suprapubic area. In the left supraclavicular area, nodular masses were palpable, raising suspicion of metastatic lymph nodes. Computed tomography (CT) revealed a huge heterogenous soft tissue mass in the pelvic cavity. Chest CT showed an extensive, conglomerated soft tissue density in the left supraclavicular area. The CA125 value was 124 u/ml. She underwent an exploratory laparotomy. At operation, a 15-cm ovarian mass was found to adhere to the uterus, bladder, rectum, and small intestine. About 500 ml of ascites was noted. The uterus, pelvic mass, and neck masses were removed, and there were large amounts of intraoperative bleeding. The pathologic diagnosis of the ovarian mass was undifferentiated non-small cell neuroendocrine carcinoma of the ovary. Immunohistochemical staining was negative for synaptophysin, but positive for chromogranin A and NSE (Figure 1). The pathologic examination revealed that the neck mass had malignant cells with massive necrosis. The cytologic evaluation of the ascites showed malignant cells. She received 1 session of etoposide 100 mg/m^2^ for 2 days along with 1 session of carboplatin 300 mg/m^2^ at 14 days after operation. She died of septic shock after 45 postoperative days.

### Case 2

A 58-year old woman was referred to our clinic after being diagnosed with neuroendocrine carcinoma of the ovary at a private clinic. She had complained of abdominal discomfort for 1 month prior to this presentation and was found to have an ovarian mass. She had no remarkable past medical history or family history. She underwent total abdominal hysterectomy with bilateral salpingo-oophorectomy, total omentectomy, and pelvic lymph node sampling at the private clinic. The pathologic diagnosis of the mass was undifferentiated non-small cell neuroendocrine carcinoma restricted to the left ovary. The surgical stage was Ia. She was treated with 6 sessions of a combination chemotherapy consisting of paclitaxel 175 mg/m^2^ and cisplatin 90 mg/m^2^. Five months after the last chemotherapy, a recurrent mass was noted around the para-aortic lymph node area by CT scan. It measured about 2.3 × 1.6 cm on the left para-aortic area. The CA125 level was 1.6 u/ml. She underwent secondary debulking operation. At the laparotomy, a 3-cm mass was noted in the left para-aortic lymph node. The mass was removed and histopathologically examined. The pathologic diagnosis of the mass was large cell neuroendocrine carcinoma recurrent in the para-aortic lymph node. She was treated with 7 sessions of taxotere 75 mg/m^2^ every 3 weeks. She died of multiple organ failure at 17 months after initial diagnosis.

### Case 3

**Figure 2:**
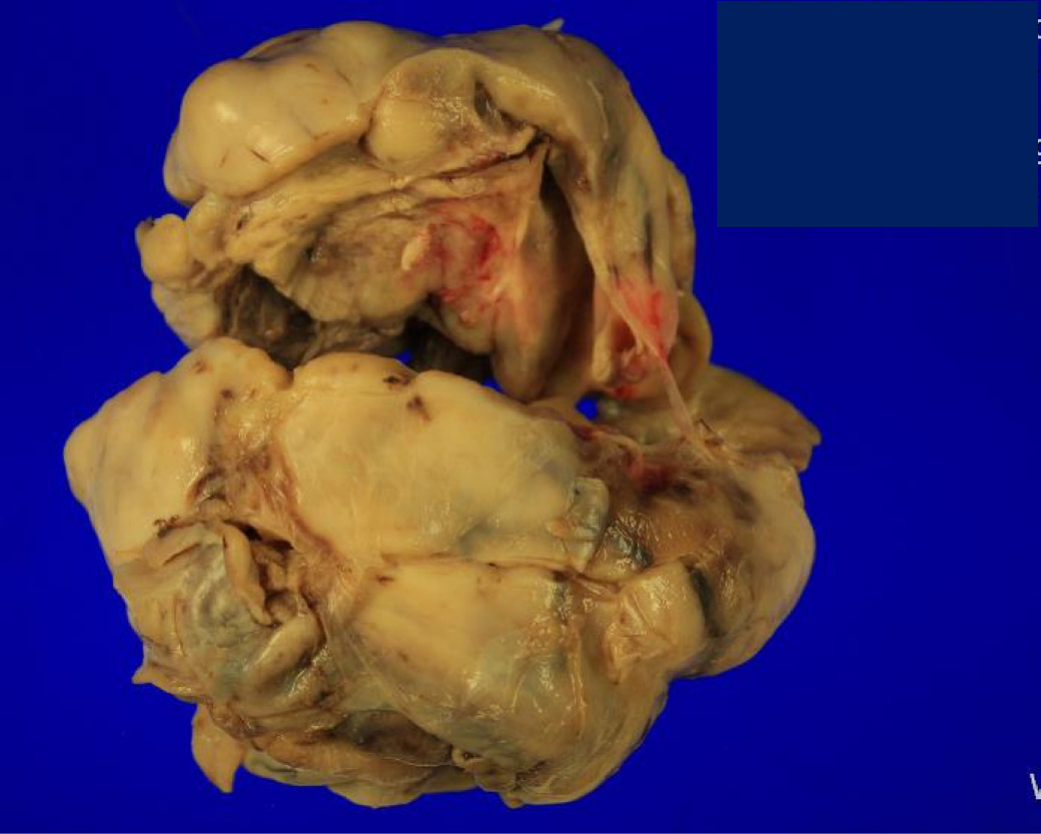
**A photograph of an ovarian mass.** It is composed of solid and cystic lesions.

A 67-year old woman was referred to our clinic because of an ovarian mass. She had visited a private clinic due to urinary frequency, and the ovarian mass was found by transvaginal sonography. At presentation, CT scan revealed a 13-cm multiseptated mixed solid and cystic mass in the left ovary which was suspected of cystadenocarcinoma. The CA125 level was 71.8 u/ml. She underwent total abdominal hysterectomy, bilateral salpingo-oophorectomy, pelvic lymph node dissection, para-aortic lymph node dissection, and total omentectomy along with multiple biopsy. At operation, the mass was composed of solid and cystic portions (Figure 2). The pathologic diagnosis of the mass was neuroendocrine carcinoma of non-small cell type arising from the left ovary which involved the pelvic peritoneum. Immunohistochemical staining was positive for pancytokeratin, CD56, and pancytokeratin, and was focally positive for NSE; however, it was negative for synaptophysin and chromogranin A (Figure 1). The surgical stage was IIb. She was treated with a combination chemotherapy consisting of paclitaxel 175 mg/m^2^ and carboplatin 4-hour area-under-the curve (AUC4) every 3 weeks. She is still healthy 5 months after operation.

## Discussion

Neuroendocrine carcinoma originates from endocrine cells of the diffuse neuroendocrine system, which in turn consists of a variety of cells present in the central and peripheral nervous systems as well as several endocrine organs. These cells can produce biologically active amines and peptides which can act as neurotransmitters, hormones, or paracrine regulators. Neuroendocrine cells are present in normal epithelium of the female genital tract [10]. It has been shown that primitive endodermal cells have the potential to differentiate into both endocrine and other cell types and that ovarian neuroendocrine tumors may develop from non-neuroendocrine cells through activation of genes that promote neuroendocrine differentiation [12].

In general, the incidence of epithelial ovarian cancer increases in older patients (age >50 years), but the LCNC can be developed in premenopausal and postmenopausal women, ranging from 22 to 76 years. Similarly, in our report the age of patients ranged from 58 to 77 years, and mean age was 67.3 years. Clinical symptoms at initial presentation are variable. The most common clinical manifestation was abdominal pain in 7 cases [3, 13], followed by abdominal distension (n = 4) [14–17], and an abdominal palpable mass (n = 3) [3, 5, 16], abdominal bloating (n = 3) [3], abdominal discomfort (n = 1) [2], postmenopausal vaginal bleeding (n = 1) [3], and dysarthria due to brain metastasis (n = 1) [8]. In our report, abdominal distension, abdominal discomfort, and urinary frequency occurred in one case each. The urinary frequency may have been due to compression of the bladder by the huge ovarian mass. Most of the LCNCs are partially solid or partially cystic, with size ranging from 9 and 30 cm (mean size, 16.6 cm) [2, 3, 8, 14, 17]. In this report, the mean size of the mass was 13 cm, and the mass was also partially solid or cystic.

The histogenesis of neuroendocrine tumors is unknown. The following hypotheses have been proposed. First, neuroendocrine cells have been presented in the normal epithelium of benign, borderline, and malignant tumors of the female genital tract. These cells serve as an origin of neuroendocrine tumors of the ovary. Second, primitive endocrine cells can differentiate into endocrine and other cell types. Third, ovarian neuroendocrine tumors may develop from non-neuroendocrine cells, which activate genes promoting neuroendocrine differentiation [12, 18].

CA125 is a tumor antigen found in 75 to 83% of all epithelial ovarian cancers [19]. Serum CA125 levels correlate with cancer stages or responses to treatment. A rise in CA125 levels usually precedes tumor progression or recurrence. Therefore, CA125 can be used to monitor epithelial ovarian cancer. In LCNC, serum CA125 levels are not specific to clinical courses. Table 1 shows reported serum CA125 levels in previous studies. The CA125 levels range from 5.7 to 917 u/ml. Some authors have reported other tumor markers. Ngan *et al.* [9] have reported that 5-hydroxyindole acetic acid (5-HIAA) markedly increase in neuroendocrine carcinomas of the ovary. They also stated that 5-HIAA is a sensitive tumor marker of neuroendocrine components.

**Table 1:** **Clinicopathologic review, adjuvant treatment, CA125 levels, and follow-up periods of reported cases**

Author	Mean age (years)	Chief complaint	Adjuvant treatment	CA125 (u/ml)	Follow-up periods (months)
Lindboe *et al*. [2]	64	Abdominal discomfort, nausea	Cisplatin + Bleomycin + Etoposide	380	9
Shakuntala *et al*. [14]	40	Abdominal distension	Cisplatin + Etoposide	280	6
Hinde *et al.* [15]	54	Abdominal distension	Carboplatin + Paclitaxcel	_	8
Dundr *et al*. [8]	73	Dysarthria, difficulty in verbal expression because of brain metastasis	Carboplatin + Paclitaxcel	94	12
			Cyberknife on brain lesion		
Tsuji *et al*. [17]	46	Abdominal distension	Carboplatin + Paclitaxcel	914	Died after 4 months postoperatively
Aslam *et al*. [13]	76	Abdominal pain	None	Within the normal range	Died postoperatively
Chenevert *et al*. [16]	53	Abdominal mass	Carboplatin + Paclitaxcel	80	Died after 3 months postoperatively
	53	Abdominal distension	Cisplatin + Etoposide	5.7	Died after 7 months postoperatively
Behnam *et al*. [5]	27	Pelvic mass	Carboplatin + Paclitaxcel		10
Veras *et al.* [3] 11 cases	22 to 63 [46.7]	6: abdominal pain			Mean survival: 12
					Mean follow-up periods: 28
		3: abdominal bloating			
		1: pelvic mass			
		1: postmenopausal bleeding			
Ki *et al.*	77				
		Abdominal distension	Carboplatin + Etoposide	124	Died after 1.5 months postoperatively
	58	Abdominal discomfort	Cisplatin + Paclitaxcel	none	Died after 17 months postoperatively
			Single Docetaxel		
	67	Urinary frequency	Carboplatin + Paclitaxcel	71.8	5

CT and magnetic resonance imaging (MRI) usually show non-specific findings in LCNC cases. Radiologic studies are not useful for the differential diagnosis of LCNC from other ovarian tumors [5, 15, 18].

The differential diagnosis of LCNC includes other primary or secondary neuroendocrine tumors. Small cell carcinoma has smaller cells with molding and necrosis. Metastatic neuroendocrine cancer cells are usually not found in the epithelial layer of the ovary. Several non-neuroendocrine tumors, such as teratoma, sex-cord stromal tumor, and Sertoli-Leydig cell tumor, may show neuroendocrine differentiation. These biphasic tumors can usually be distinguished from LCNC by identifying non-neuroendocrine components [3, 5, 12]. Immunohistochemistry is important to diagnose neuroendocrine carcinoma. The most commonly used non-hormonal immunohistochemical markers are chromogranin A, synaptophysin, cytokeratin, and CD56. NSE and Leu-7 lack specificity and may not be conclusive for neuroendocrine differentiation when other stains are negative [3, 5, 15, 16, 18]. Our specimens were immunohistochemically positive for chromogranin A, NSE, CD56, and pancytokeratin.

There is no standard treatment of LCNC. Various combination chemotherapy regimens, such as platinum, paclitaxcel, etoposide, and bleomycin have been used in previous studies. The survival periods varied among the groups. A combination of platinum and paclitaxcel has most frequently been used with shorter survival.

## Conclusion

LCNC is rare, shows aggressive behaviors and poor responses to treatment. Due to the rarity of LCNCs, general consensus on the standard therapy has not yet been established. Although patients with LCNC are at stage I, their survival rates are relatively low due to biological aggressiveness despite extensive surgery and chemotherapy. We reported three cases of advanced or early LCNC with a brief review of the literature.

## Consent

Written informed consent was obtained. The study was approved by the Institutional Review Board of our hospital (KC14ZISE0113).

## Abbreviations

5-HIAA: 5-hydroxyindole acetic acid
AUC4: 4-hour area-under-the curve
CT: computed tomography
H&E: hematoxylin and eosin
LCNC: large cell neuroendocrine carcinoma
MRI: magnetic resonance image
WHO: World Health Organization.
